# Factors Associated With Use of the Preventive Health Inventory in US Veterans

**DOI:** 10.1001/jamanetworkopen.2024.2717

**Published:** 2024-03-18

**Authors:** Chelle L. Wheat, Edwin S. Wong, Kristen E. Gray, Susan E. Stockdale, Karin M. Nelson, Ashok Reddy

**Affiliations:** 1 Center for Veteran-Centered and Value-Driven Care, Veterans Affairs (VA) Puget Sound Health Care System, Seattle, Washington; 2 Department of Health Systems and Population Health, University of Washington, Seattle; 3 Division of General Internal Medicine, Department of Medicine, University of Washington, Seattle; 4 VA Greater Los Angeles Healthcare System, Center for the Study of Healthcare Innovation, Implementation and Policy (CSHIIP), Los Angeles, California; 5 David Geffen School of Medicine, Department of Medicine, Division of General Internal Medicine, University of California at Los Angeles, Los Angeles

## Abstract

**Importance:**

The COVID-19 pandemic caused significant declines in the quality of preventive and chronic disease care. The Veterans Health Administration (VHA) used the Preventive Health Inventory (PHI), a multicomponent care management intervention, to catch up on care disrupted by the pandemic.

**Objective:**

To identify key factors associated with PHI use.

**Design, Setting, and Participants:**

This cohort study of veterans receiving primary care used administrative data from national VHA primary care clinics for February 1, 2021, through February 1, 2022.

**Exposure:**

Patient PHI receipt.

**Main Outcomes and Measures:**

The main outcomes were patient, practitioner, and clinic factors associated with PHI receipt. Binomial generalized linear models with fixed effects for clinic were used to analyze factors associated with receipt of PHI. Least absolute shrinkage and selection operator procedures were used for variable selection.

**Results:**

A total of 4 358 038 veterans (mean [SD] age, 63.7 [16.0] years; 90% male; 76% non-Hispanic White) formed the study cohort, of whom 389 757 (9%) received the PHI. Veterans who received the PHI had higher mean Care Assessment Need (CAN) scores, which indicate the likelihood of hospitalization or death within 1 year (mean [SD], 51.9 [28.6] vs 47.2 [28.6]; standardized mean difference [SMD], −0.16). They were also more likely to live in urban areas (77% vs 64%; SMD, 0.28) and have a shorter drive distance to primary care (mean [SD], 13.2 [12.4] vs 15.7 [14.6] miles; SMD, 0.19). The mean outpatient use was higher among PHI recipients compared with non-PHI recipients (mean [SD], 18.4 [27.8] vs 15.1 [24.1] visits; SMD, −0.13). In addition, veterans with primary care practitioners with higher caseloads were more likely to receive the PHI (mean [SD], 778 [231] vs 744 [249] patients; SMD, −0.14), and they were more likely to be seen at larger clinics (mean [SD], 9670 [6876] vs 8786 [6892] patients; SMD, −0.13). Prior outpatient use and CAN score were associated with PHI receipt in the final model.

**Conclusions and Relevance:**

In this cohort study of the VHA’s PHI, patients with higher CAN scores and more outpatient use in the previous year were more likely to receive the PHI. This study identifies potential intervention points to improve care coordination for veterans.

## Key Points

**Question:**

Which key patient, practitioner, and clinical characteristics are associated with Preventive Health Inventory (PHI) use?

**Findings:**

In this cohort study of more than 4.3 million veterans, of whom 9.0% received the PHI, patients with Care Assessment Need scores and more outpatient use in the prior year were more likely to receive the PHI.

**Meaning:**

These findings suggest that targeted outreach to veterans who use fewer primary care services may be needed to ensure that they receive necessary chronic disease management and preventive care.

## Teaser

This cohort study examines patient-, practitioner-, and clinic-level factors associated with receipt of the Preventive Health Inventory among US veterans.

## Introduction

In response to the COVID-19 pandemic, the Veterans Health Administration's (VHA’s) Office of Primary Care developed the Preventive Health Inventory (PHI) program in February 2021^1^ to catch up on delayed or disrupted care This multicomponent care management intervention included development of a national dashboard of quality measures, telehealth appointments with a nurse, and completion of a templated electronic health record note that comprises a checklist of care needs. The nurse visit and use of the templated note included screening and management for mental health, cancer prevention, and chronic disease management (eTable in Supplement 1).^1^ Prior evaluations have not examined veteran receipt of PHI, a critical gap because the program targets specific chronic and preventive care needs of veterans. Understanding individual characteristics that influence PHI receipt is crucial to identify any differences in care based on specific patient, practitioner, and clinic factors. Our goal was to examine differences in individual characteristics and factors associated with PHI receipt to help the VHA address any barriers to receiving the PHI care.

## Methods

We conducted a retrospective cohort study of veterans receiving primary care at the VHA between February 1, 2021, and February 1, 2022. We obtained all data from the VHA’s Corporate Data Warehouse, a national repository of clinical and administrative data. To assess PHI use, we extracted specific administrative data created to track PHI template use. This study followed the Strengthening the Reporting of Observational Studies in Epidemiology (STROBE) reporting guideline. This study was approved by the Veterans Affairs National Institutional Review Board. The institutional review board waived the need for consent because the study is considered minimal risk.

The following patient, practitioner, and clinic factors were examined in relation to their association with PHI receipt: age (years), sex (male or female), race and ethnicity (Alaska Native or Native American; Asian, Pacific Islander, or Native Hawaiian; Hispanic; non-Hispanic Black; or non-Hispanic White),^2^ marital status, priority status,^3^ neighborhood socioeconomic status (decile based on census data) as a surrogate marker for income,^4^ rurality of residence (urban, rural, or highly rural or insular islands), drive distance to primary care (miles), Gagne comorbidity score (scores range from <0 to >9, with increased scores corresponding to increased risk of 1-year mortality),^5^ Care Assessment Need (CAN) score (defined as the probability of hospital admission or mortality within 1 year, converted to a percentile),^6^ count of outpatient visits in the prior year, and inpatient use in the prior year. Race and ethnicity were included as a factor in this study because there is evidence that telehealth interventions may be used less among those who identify as being in a racial or ethnic minority group. All data were extracted from administrative databases derived from the electronic health record and patient experience surveys. Practitioner variables (veteran assigned) included panel fullness (number of primary care patients cared for, adjusted for full-time equivalents), practitioner type (doctor of medicine, doctor of osteopathic medicine, physician assistant, or nurse practitioner), role of person completing the PHI reminder (physician or nurse), full-time equivalents, age (years), sex (male or female), and years of VHA tenure. Clinic variables included total clinic size (number of enrolled patients at site), staffing ratio (number of support staff for each practitioner), facility type (VHA medical center or outpatient clinic), geographic regional system of care (Veterans Integrated Service Network), and rurality (urban, rural, or highly rural or insular islands).

### Statistical Analysis

We used bivariate analyses to calculate standardized mean differences (SMDs) comparing patient, practitioner, and clinic characteristics between veterans who did and did not receive the PHI. Standardized mean differences less than 0.1 were considered meaningful.^7^ We used binomial generalized linear models with fixed effects for clinics to estimate the association of the variables of interest and receipt of PHI. Receipt of PHI was defined as evidence of a completed templated clinic note in the veteran’s electronic health record. To facilitate variable selection, we used least absolute shrinkage and selection operator procedures. For inference, we calculated marginal effects for each explanatory variable, using the Δ method for SEs. Estimated marginal effects represent differences on the probability scale. All analyses were performed using R, version 3.4.1 (R Foundation for Statistical Computing).

## Results

Table 1 gives the characteristics of 4 358 038 veterans in primary care, of whom 389 757 (9%) received the PHI. Overall, the mean (SD) age was 63.7 (16.0) years, 3 908 034 veterans (90%) were male, and 450 004 (10%) were female. Among 4 051 965 with available race and ethnicity, 36 982 (1%) were American Indian or Alaska Native; 90 036 (2%) were Asian, Pacific Islander, or Native Hawaiian; 76 913 (2%) were Hispanic; 780 617 (19%) were non-Hispanic Black; and 3 067 417 (76%) were non-Hispanic White. The SMDs across the groups were similar for patient characteristics, except PHI receipt was higher in those with higher CAN scores (mean [SD], 51.9 [28.6] vs 47.2 [28.6]; SMD, −0.16), in veterans with higher Gagne comorbidity score (mean [SD], 0.61 [1.64] vs 0.52 [1.56]; SMD, −0.06), in veterans living in urban areas (77% vs 64%; SMD, 0.28), in those with shorter driving distance (mean [SD], 13.2 [12.4] vs 15.7 [14.6] miles; SMD, 0.19), and in those who received more outpatient visits (mean [SD], 18.4 [27.8] vs 15.1 [24.1]; SMD, −0.13).

**Table 1.** Unadjusted Associations Between Potential Factors Associated With PHI Use

Characteristic	No./total No. (%)			SMD (95% CI)
	Overall (N = 4 358 038)	Did not receive PHI (n = 3 968 281)	Received PHI (n = 389 757)	
**Veterans **				
Age, mean (SD), y	63.7 (16.0)	63.6 (16.1)	65.0 (15.5)	−0.09 (−0.09 to −0.08)
Sex				
Male	3 908 034/4 358 038 (90)	3 558 253/3 968 281 (90)	349 781/389 757 (90)	0.00 (0.00 to 0.01)
Female	450 004/4 358 038 (10)	410 028/3 968 281 (10)	39 976/389 757 (10)	
Race and ethnicity				
American Indian or Alaska Native	36 982/4 051 965 (1)	34 560/3 689 568 (1)	2422/362 397 (1)	0.08 (0.08 to 0.08)
Asian, Pacific Islander, or Native Hawaiian	90 036/4 051 965 (2)	81 581/3 689 568 (2)	8455/362 397 (2)	
Hispanic	76 913/4 051 965 (2)	66 586/3 689 568 (2)	10 327/362 397 (3)	
Non-Hispanic Black	780 617/4 051 965 (19)	714 548/3 689 568 (19)	66 069/362 397 (18)	
Non-Hispanic White	3 067 417/4 051 965 (76)	2 792 293/3 689 568 (76)	275 124/362 397 (76)	
Married	3 046 438/4 358 038 (70)	2 779 324/3 968 281 (70)	267 114/389 757 (69)	0.03 (0.03 to 0.04)
Priority status				
1-3	2 735 426/4 335 203 (63)	2 495 165/3 947 235 (63)	240 261/387 968 (62)	0.03 (0.02 to 0.03)
4-6	931 692/4 335 203 (21)	844 711/3 947 235 (21)	86 981/387 968 (22)	
7-8	668 085/4 335 203 (15)	607 359/3 947 235 (15)	60 726/387 968 (16)	
Gagne comorbidity score, mean (SD)	0.52 (1.57)	0.52 (1.57)	0.61 (1.64)	−0.06 (−0.06 to −0.05)
CAN score, mean (SD)	47.64 (28.65)	47.23 (28.62)	51.86 (28.58)	−0.16 (−0.17 to −0.16)
Neighborhood SES index (decile)				
0	293 419/4 294 688 (6.8)	259 398/3 907 889 (6.6)	34 021/386 799 (8.8)	0.09 (0.08 to 0.09)
1	374 734/4 294 688 (8.7)	340 853/3 907 889 (8.7)	33 881/386 799 (8.8)	
2	441 480/4 294 688 (10)	401 738/3 907 889 (10)	39 742/386 799 (10	
3	486 647/4 294 688 (11)	444 346/3 907 889 (11)	42 301/386 799 (11)	
4	498 114/4 294 688 (12)	451 610/3 907 889 (12)	46 504/386 799 (12)	
5	507 526/4 294 688 (12)	462 971/3 907 889 (12)	44 555/386 799 (12)	
6	495 693/4 294 688 (12)	452 763/3 907 889 (12)	42 930/386 799 (11)	
7	483 293/4 294 688 (11)	441 999/3 907 889 (11)	41 294/386 799 (11)	
8	420 126/4 294 688 (9.8)	382 634/3 907 889 (9.8)	37 492/386 799 (9.7)	
9	276 201/4 294 688 (6.4)	253 525/3 907 889 (6.5)	22 676/386 799 (5.9)	
Rurality				
Urban	2 841 596/4 333 348 (66)	2 543 432/3 945 493 (64)	298 164/387 855 (77)	0.28 (0.27 to 0.28)
Rural	1 322 339/4 333 348 (31)	1 240 885/3 945 493 (31)	81 454/387 855 (21)	
Highly rural/insular islands	169 413/4 333 348 (3.9)	161 176/3 945 493 (4.1)	8237/387 855 (2.1)	
Drive distance to primary care, mean (SD), m	15.50 (14.40)	15.73 (14.56)	13.21 (12.35)	0.19 (0.18 to 0.19)
No. of outpatient visits in past year, mean (SD)	15.41 (24.47)	15.11 (24.10)	18.40 (27.78)	−0.13 (−0.13 to −0.12)
No. of hospitalizations in past year, mean (SD)	0.07 (0.43)	0.07 (0.43)	0.09 (0.49)	−0.05 (−0.05 to −0.05)
**Practitioners**				
Panel size, mean (SD)	746.86 (247.48)	743.77 (248.80)	778.29 (231.25)	−0.14 (−0.15 to −0.14)
Panel fullness, mean (SD)	0.76 (0.15)	0.76 (0.15)	0.76 (0.14)	0.04 (0.03 to 0.04)
Practitioner type				
Doctor of medicine	2 298 213/3 077 607 (75)	2 061 946/2 791 575 (74)	236 267/286 032 (83)	0.23 (0.22 to 0.23)
Nurse practitioner or physician assistant	665 944/3 077 607 (22)	626 354/2 791 575 (22)	39 590/286 032 (14)	
Other	113 450/3 077 607 (3.7)	103 275/2 791 575 (3.7)	10 175/286 032 (3.6)	
Full-time equivalents, mean (SD)	0.90 (0.84)	0.89 (0.88)	0.92 (0.18)	−0.04 (−0.04 to −0.03)
Age, mean (SD), y	54.07 (9.70)	54.05 (9.72)	54.22 (9.46)	−0.02 (−0.02 to −0.01)
Sex				
Female	1 358 030/2 355 290 (58)	1 228 483/2 127 236 (58)	129 547/228 054 (57)	0.02 (0.01 to 0.02)
Male	997 260/2 355 290 (42)	898 753/2 355 290 (42)	98 507/2 355 290 (43)	
VA tenure, y	13.99 (8.21)	13.99 (8.22)	13.97 (8.09)	0.00 (0.00 to 0.01)
**Clinics **				
Clinic size, mean (SD)	8865.40 (6894.79)	8785.98 (6891.60)	9670.29 (6875.47)	−0.13 (−0.13 to −0.13)
Staffing ratio, mean (SD)	3.66 (3.06)	3.69 (3.18)	3.35 (1.41)	0.14 (0.14 to 0.15)
Clinic type				
Community-based outpatient clinic	2 850 277/4 358 038 (65)	2 600 066/3 968 281 (66)	250 211/389 757 (64)	0.03 (0.02 to 0.03)
VA medical center	1 507 761/4 358 038 (35)	1 368 215/3 968 281 (34)	139 546/389 757 (36)	
Rurality				
Urban	3 569 077/4 358 038 (82)	3 224 430/3 968 281 (81)	344 647/389 757 (88)	0.20 (0.20 to 0.20)
Rural	788 961/4 358 038 (18)	743 851/3 968 281 (19)	45 110/389 757 (12)	

Abbreviations: CAN, Care Assessment Needs; PHI, Preventive Health Inventory; SES, socioeconomic status; SMD, standardized mean difference; VA, Veterans Affairs.

Veterans who received the PHI had primary care practitioners with higher panel fullness (mean [SD], 778 [231] vs 744 [249]; SMD, −0.14) and attended larger clinics (mean [SD], 9670 [6876] vs 8786 [6892]; SMD, −0.13). Veterans who received the PHI had primary care practitioners with lower staffing ratios (mean [SD], 3.4 [1.4] vs 3.7 [3.2]; SMD, −0.14) and were more likely to attend primary care clinics in an urban location (88% vs 81%; SMD, 0.20).

The estimated marginal effect for the CAN score was that those in the highest tertile (score of 70-99) had a 2.08% (95% CI, 1.64%-2.51%) probability of PHI receipt compared with those that did not receive the PHI (Table 2). Those in the middle tertile (CAN score of 50-69) had a 1.06% (95% CI, 0.81%-1.31%) probability of PHI receipt, whereas those in the lowest tertile (CAN score <50) had a 0.54% (95% CI, 0.37%-0.70%) probability of PHI receipt. The estimated marginal effect for outpatient use was a 1.48% (95% CI, 1.02%-1.93%) probability of PHI receipt compared with those who did not receive the PHI for patients in the highest tertile (≥19 visits), 0.85% (95% CI, 0.56%-1.15%) for those in the middle tertile (8-18 visits), and 0.34% (95% CI, 0.13%-0.54%) for those in the lowest tertile (0-7 visits).

**Table 2.** Average Marginal Effects for Factors Associated With Preventive Health Inventory Use

Factor	Average marginal effect, % (95% CI)^a^
Outpatient use	
≥19 visits	1.48 (1.02 to 1.93)
8-18 visits	0.85 (0.56 to 1.15)
0-7 visits	0.34 (0.13 to 0.54)
CAN score	
70-99	2.08 (1.64 to 2.51)
50-69	1.06 (0.81 to 1.31)
<50	0.54 (0.37 to 0.70)
Drive distance to primary care, m	−0.05 (−0.11 to 0.01)
Panel fullness	1.53 (−1.76 to 4.82)

Abbreviation: CAN, Care Assessment Needs.

^a^ Calculated as the average of all the observation-specific marginal effects.

## Discussion

We found that patients with higher CAN scores and more outpatient use in the previous year were more likely to receive the PHI. These findings suggest that patients receiving more care are more likely to receive the PHI, perhaps due to the increased opportunities for the patient to access this care and higher care needs.^8^ The finding that PHI receipt was positively associated with higher CAN scores differs from prior findings of preventive care and comorbidity burden.^9,10^ However, there is evidence that individuals with a higher number of comorbidities have higher health care use, especially primary care.^11,12^ This finding may support the idea that PHI delivery is targeted to veterans living with more comorbidities who needed care.

### Limitations

Limitations include the use of VHA administrative data, which may miss care that is captured outside the VHA. Moreover, as in all observational studies, unmeasured confounding can limit findings. Also, this work may not be generalizable outside the VHA and integrated into the VHA’s electronic health record system.

## Conclusions

This cohort study offers valuable insights to improve care coordination for veterans. The PHI was developed as a telehealth intervention to catch up on care disrupted by the COVID-19 pandemic; however, this intervention can now be used to target veterans at need. As the PHI expands, support may be needed to help clinics deliver the intervention to patients who use fewer health care services, experience digital barriers, or live in rural communities. Future research should examine whether using the PHI with patients results in better quality of preventive care as well as explore who practitioners are choosing to target for administering the PHI.

## Notes

Supplement 1. **eTable.** Description of PHI Services

Supplement 2.
Data Sharing Statement
