# Supplementary material for: Factors Associated With Use of the Preventive Health Inventory in US Veterans
Source: JAMA Netw Open. 2024 Mar 18;7(3):e242717. doi: 10.1001/jamanetworkopen.2024.2717 (PMC10949100; doi:10.1001/jamanetworkopen.2024.2717)
Supplement: Supplement 1. — eTable. Description of PHI Services [file jamanetwopen-e242717-s001.pdf]

Supplementary Online Content

Wheat CL, Wong ES, Gray KE, et al. Factors associated with use of the preventive health inventory in US veterans. *JAMA Netw Open*. 2024;7(3):e242717. doi:10.1001/jamanetworkopen.2024.2717

eTable. Description of PHI Services

This supplementary material has been provided by the authors to give readers additional information about their work.

© 2024 Wheat CL et al. *JAMA Network Open.*

eTable. Description of PHI Services

Service Type	Preventive Screening	Chronic Condition Monitoring
**Counseling**	• Blood pressure<br>• Influenza vaccine<br>• Colorectal cancer screening<br>• Breast cancer screening<br>• Cervical cancer screening<br>• Diabetes	• Blood pressure<br>• Diabetes
**Point of care (Completed)**	• Home blood pressure<br>• Suicide risk screening	• Home blood pressure<br>• Diabetes foot exam (modified video exam or reported)
**Orders (Planned)**	• Influenza vaccine<br>• Colorectal cancer screening<br>• Breast cancer screening<br>• Cervical cancer screening (visit scheduled)	• Hemoglobin A1c<br>• Microalbumin<br>• Diabetes foot exam (visit scheduled)<br>• Diabetic retinopathy screening/ monitoring (visit scheduled)

© 2024 Wheat CL et al. *JAMA Network Open.*
