# Supplementary material for: Factors Associated With Use of the Preventive Health Inventory in US Veterans
Source: JAMA Netw Open. 2024 Mar 18;7(3):e242717. doi: 10.1001/jamanetworkopen.2024.2717 (PMC10949100; doi:10.1001/jamanetworkopen.2024.2717)
Supplement: Supplement 2. — Data Sharing Statement [file jamanetwopen-e242717-s002.pdf]

# Data Sharing Statement

Wheat. Factors Associated With Use of the Preventive Health Inventory in US Veterans. *JAMA Netw Open*. Published March 18, 2024. doi:10.1001/jamanetworkopen.2024.2717

**Data**
**Data available:** No
